# Combining education and income into a socioeconomic position score for use in studies of health inequalities

**DOI:** 10.1186/s12889-022-13366-8

**Published:** 2022-05-13

**Authors:** Marie Hella Lindberg, Gang Chen, Jan Abel Olsen, Birgit Abelsen

**Affiliations:** 1 Department of Community Medicine, UiT – The Arctic University of Norway, 9037 Tromsø, Norway; 2 Centre for Health Economics, Monash University, Melbourne, Australia; 3 Division of Health Services, Norwegian Institute of Public Health, Oslo, Norway

**Keywords:** Socioeconomic position, Socioeconomic status, Health inequalities, Health-related quality of life, Composite indicator

## Abstract

**Background:**

In studies of social inequalities in health, there is no consensus on the best measure of socioeconomic position (SEP). Moreover, subjective indicators are increasingly used to measure SEP. The aim of this paper was to develop a composite score for SEP based on weighted combinations of education and income in estimating subjective SEP, and examine how this score performs in predicting inequalities in health-related quality of life (HRQoL).

**Methods:**

We used data from a comprehensive health survey from Northern Norway, conducted in 2015/16 (*N* = 21,083). A composite SEP score was developed using adjacent-category logistic regression of subjective SEP as a function of four education and four household income levels. Weights were derived based on these indicators’ coefficients in explaining variations in respondents’ subjective SEP. The composite SEP score was further applied to predict inequalities in HRQoL, measured by the EQ-5D and a visual analogue scale.

**Results:**

Education seemed to influence SEP the most, while income added weight primarily for the highest income category. The weights demonstrated clear non-linearities, with large jumps from the middle to the higher SEP score levels. Analyses of the composite SEP score indicated a clear social gradient in both HRQoL measures.

**Conclusions:**

We provide new insights into the relative contribution of education and income as sources of SEP, both separately and in combination. Combining education and income into a composite SEP score produces more comprehensive estimates of the social gradient in health. A similar approach can be applied in any cohort study that includes education and income data.

**Supplementary Information:**

The online version contains supplementary material available at 10.1186/s12889-022-13366-8.

## Background

An extensive empirical literature has documented a positive association between individuals’ socioeconomic position (SEP) and their health, commonly referred to as the social gradient in health [1, 2]. The social gradient reflects that individuals’ structural location in society is an important determinant of the likelihood of experiencing health-damaging exposures, or of holding certain health-enhancing resources [3]. This is largely built on the theoretical contribution of Max Weber, who argued that society is stratified into hierarchies along various dimensions, creating groups based on different sets of skills, knowledge, and assets. These factors, which Weber defined as individuals’ “life chances”, produce social stratification and will, as such, determine individuals’ position in the marketplace [4]. Measures of SEP aim to reflect these life chances [5].

However, there is no single measure that best identifies SEP [4]. Therefore, SEP is most commonly captured by three proxy measures: education, occupation and/or income [6]. Through various mechanisms, these measures produce status that is considered health-enhancing (see e.g., Marmot [7]). While closely related, the three measures are not interchangeable [8, 9].

A growing literature suggests that *subjective* SEP measures are also powerful determinants of health [10]. Rather than focusing solely on objective indicators of SEP, inequalities in subjective SEP could be as important, or even more strongly linked to health than objective SEP measures [11, 12]. This builds on the hypothesis that subjective SEP captures socioeconomic dimensions not measured by objective SEP indicators [13, 14]. For example, in The English Longitudinal Study of Ageing, it was found that subjective SEP mediated the association between objective SEP measures and mortality, as well as independently predicting mortality [10]. Additionally, none of the objective SEP indicators directly measure socially derived attributions of prestige or status [15]. This suggests that objective SEP measures should be complemented by a measure of subjective SEP.

The association between SEP and health has been observed with each of the three objective SEP indicators, which could indicate that SEP represents a broader, underlying construct related to social stratification [16]. Therefore, if these SEP variables capture different aspects of the same concept [6], a composite measure could better represent SEP when estimating social inequalities in health [17]. Additionally, a composite SEP measure may capture multiple aspects of relevance when estimating how individuals’ SEP influences health inequalities, thus simplifying interpretation [18] and communication of results [19].

In the literature on social inequalities in health, composite indicators of SEP are applied in different ways. The focus here will be on individual-level composite indicators. Early examples include the Hollingshead index of social status [20], using a priori defined weights for education and occupation; the Duncan’s socioeconomic index for occupational prestige; and the Nam-Powers occupational status score [19, 21]. These indicators are not as relevant today due to changes in eduation and the labour market [22]. However, the Nam-Powers score has in later years been updated and refined into the Nam-Powers-Boyd occupational score, using data from the 2010–12 American Community Surveys, in which median education and median earnings of different occupations are used as the basis for the score [23]. In the UK, occupation is widely used for socioeconomic classifications [6]. In application today is the National Statistics Socio-economic classification, which incorporates employment relations and conditions of occupations, into non-hierarchical occupational classes [24]. The latter two examples are limited to the US and UK contexts, and would need to be adjusted to fit other contexts. Other recent examples of composite SEP indicators most frequently use education, occupation and income for composite SEP indices (see e.g., [25, 26]), as well as education and income only (e.g., [27, 28]).

A common critique against composite SEP measures is that they conceal the relative influence of their components [29]. However, it can also be argued that a composite indicator of SEP can capture the synergies between its different components [17]. In this paper, we propose a composite SEP score that compiles several SEP indicators into one, that still allows for disaggregation of the score’s components.

In the literature on social inequalities, the most common health indicators are mortality or disease-specific health outcomes (e.g., [30–32]), or self-rated health [33]. In this paper, we use two measures of health-related quality of life (HRQoL): the multidimensional EQ-5D-5L descriptive system, and a visual analogue scale (VAS).

The current paper is based on data from a general adult population and aims to: i) develop a composite SEP score from empirically derived weights that reflect individuals’ subjective SEP; and ii) test how the composite SEP score predicts inequalities in HRQoL. We regress subjective SEP on education and income levels. The resulting weights are used to predict a SEP value for each individual based on combinations of their education and income levels. We further demonstrate how the composite SEP score predicts inequalities in HRQoL. This study contributes to the literature by proposing a simple composite SEP score based on the two most widely collected objective indicators of SEP using derived weights according to their influence on subjective SEP.

### Conceptual framework

The concept of SEP is complex. It is therefore necessary to describe its components and the hypothesised relationships between them.

Education proxies an individual’s cognitive resources and the ability to process health information [4]. In addition, education has been found to be strongly associated with childhood socieconomic conditions (see e.g., [34]), and can, as such, be understood as a representation of early-life circumstances. Education is often measured as the highest level of educational attainment, or as years of education.

Occupation mirrors educational achievement, yields income, and reflects individuals’ social standing [35]. Occupation indicators can capture the prestige associated with specific professions; environmental exposures on the job (e.g., pollution); or psychosocial aspects, such as job strain and satisfaction [6].

Income is hypothesised to impact health through the ways in which individuals’ resources provide a healthy physical environment, healthier lifestyle and/or ease of access to health services. Additionally, income itself can entail a higher SEP [6].

**Fig. 1:**
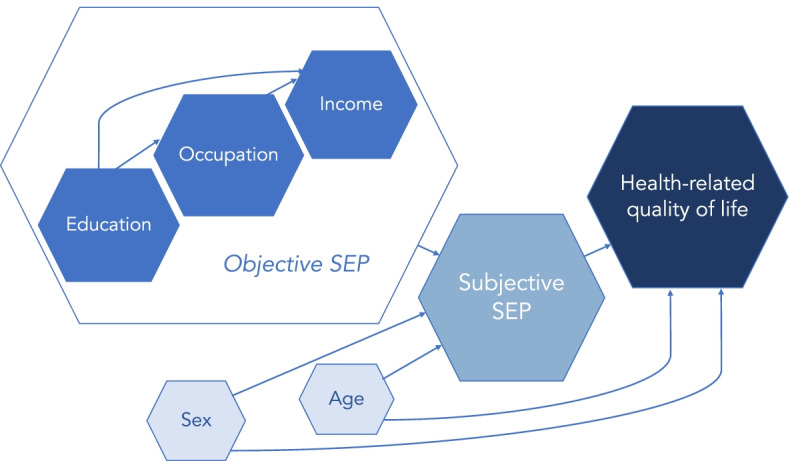
Conceptual framework of the relationship between the components of SEP and HRQoL. Note: Arrows reflect hypothesised associations between key concepts. SEP: socioeconomic position

These three indicators can be conceptualised as components of the latent construct of objective SEP. This is shown in our conceptual framework (Fig. 1), which demonstrates the hypothesised links between the key concepts included in this paper. Education provides skills and knowledge that qualify people for specific occupations. The higher education level that an occupation requires, the more cognitive resources and skills does the individual possess, all of which are associated with objective SEP. However, occupations with similar levels of educational attainment (e.g., a physician vs a priest) differ immensely in terms of income levels: having a high income, then, reflects that the individual has an occupation that society values more highly. Thus, education, occupation, and income represent the concept of objective SEP, displayed in Fig. 1, as encompassing these three indicators. In this framework, objective SEP predicts subjective SEP, which in turn, determines HRQoL. Additionally, age and sex are added as covariates, as they are assumed to influence both subjective SEP and HRQoL. They will also likely influence objective SEP, but this model focuses on how they relate to subjective SEP. Lastly, although not included in this paper, it is important to acknowledge the role played by the intergenerational transmission of both socioeconomic factors and health. It is widely established that parents not only transfer their genes to their offspring, but also their SEP and health (behaviours) [36–38].

In the current study, occupational category is not directly included in the composite SEP score. However, it is indirectly captured, in that occupation (to a large extent) is determined by education, *and* (to an even larger extent) a determinant of income. As opposed to education, measured in years; and income, measured in money, occupational categories can be more difficult to hierarchically order. This is because the categories include individuals with large differences in skills, prestige, power, and/or incomes, and are arguably not originally developed as a SEP measure [8]. Moreover, occupational measures vary widely in what they proxy and are likely to differ substantially between countries and different contexts [4]. In this sense, education and income are more consistently available from surveys and registers than occupation.

## Methods

### Data

The Tromsø Study is a prospective cohort study from a general adult population residing in the municipality of Tromsø. With approximately 77,000 inhabitants, Tromsø is the largest city in Northern Norway. The current paper is based on data from the seventh wave conducted in 2015/16. Of the 32,591 people that were invited (aged 40 years and older), *N* = 21,083 (65%) completed the survey. The study design is described in detail elsewhere [39].

The study was approved by the Regional Committee for Medical Research Ethics Northern Norway (REK North; ID 2019/607). The Tromsø Study complies with the Declaration of Helsinki and all participants gave written informed consent before admission. Data access was granted by the Data and Publication Committee of the Tromsø Study. All methods were carried out in accordance with relevant guidelines and regulations.

### Variables

Education was recorded as the highest completed education level, categorised into four: primary education up to ten years; upper secondary and vocational school; undergraduate (less than four years of higher education); and postgraduate degree (four years or more of higher education).

Income was recorded as the combined gross income of adults in the household, in eight income brackets. These were collapsed to approximate quartiles. Income groups were (per NOK 1,000): Low: ≤ NOK 450 (20.9%); Lower middle: NOK 451–750 (29.3%); Upper middle: NOK 751–999 (24.2%); and High: NOK ≥ 1 million (25.6%).

Inspired by the seminal work of Marmot on the crucial role of social status [7], we used subjective SEP to develop the composite SEP score. Subjective SEP was obtained from the statement ‘I consider my occupation to have the following social status (if you are currently out of work, think about your latest occupation)’, which was rated using a five-level scale (very high; fairly high; middle; fairly low; very low). With few respondents in the lowest category (< 1%), we collapsed the bottom one into the category for ‘low’ status, leaving subjective SEP as a four-level ordinal variable. The subjective SEP measure is framed in terms of the perceived SEP of respondents’ *occupation*, as an individual’s occupation is thought to largely shape the perception of own social standing. It is a variant of the more commonly applied MacArthur scale of subjective social status [11].

HRQoL was the main outcome variable and was measured in two ways: directly on a VAS, and indirectly with the EuroQol EQ-5D. The EQ-5D is the most widely applied generic preference-based descriptive system [40, 41]. It describes health along five dimensions: mobility, self-care, usual activities, pain/discomfort, and anxiety/depression [42]. We applied the most recent version with five severity levels along each dimension (EQ-5D-5L) [43]: ‘no problems’, ‘slight problems’, ‘moderate problems’, ‘severe problems’, and ‘unable’. In the absence of a Scandinavian value set for the EQ-5D, we used an amalgam tariff, the Western preference pattern (WePP), representing a hybrid of four Western countries’ published value sets [44]. The VAS asks respondents to rate their health today on a scale from [0–100]. The VAS was converted into a [0–1] interval for reasonable comparison with the EQ-5D value.

Age and sex were included as covariates.

### Statistical analyses

#### Descriptive statistics

Means, proportions, and standard deviations (SD) of the included variables were reported for the full sample and stratified by sex. We excluded respondents above the age of 80 (N = 761) due to a disproportionately low response rate and to diminish the impact of cohort effects on the education variable, leaving a sample of *N* = 20,322. For the analyses, respondents with missing observations for education and income were excluded, corresponding to 4.5% of the sample.

#### Regression-based approach to develop a composite SEP score

To develop the composite SEP score, we applied subjective SEP as the dependent variable, proxying SEP. We used adjacent-category logistic regression, which is an alternative to classical ordered logistic regression. This method compares each category (level) of the dependent variable with the next larger response category [45]. We modelled the four-level subjective SEP ($sSEP$) variable as a function of education ($Educ$), and income ($Inc$) (Eq. 1). The education and income variables were dummy-coded, with the lowest level serving as the reference level for each variable. Sex and age (in years) were included as control variables ($X$):

1 $$sSEP=f\left(Educ,Inc,X\right)$$

The resulting regression coefficients from Eq. 1 were used as education and income weights in the composite SEP score. Each of the education and income levels was multiplied with their corresponding regression coefficient, resulting in a composite SEP score that predicts individuals’ SEP, demonstrated in Eq. 2:

2 $${SEP}_{i}={\sum }_{j=1}^{k}{\beta }_{j}*{Educ}_{ij}+{\sum }_{j=1}^{k}{\gamma }_{j}*{Inc}_{ij}$$

This approach was inspired by Mehta et al. [46]: instead of using the risk ratios to construct a summary score, the composite SEP score was generated based on the regression coefficients modelled in Eq. 1. The SEP summary score from Eq. 2 was rescaled into a [1–10] interval to form the composite SEP score: first, the coefficients for all combinations of education and income level $j$ were added together. Second, each value of the composite SEP score was rounded to the nearest integer, resulting in a predicted SEP value [1–10] for each individual $i$ based on their combinations of income and education levels. As such, we identified how different levels of education and income influence subjective SEP.

#### Predicting variation in HRQoL

To evaluate how the composite SEP score predicted variation in HRQoL (EQ-5D and VAS), we ran ordinary least squares (OLS) regression of HRQoL on the composite SEP score, adjusted for age and sex. Further, we calculated the age-adjusted predicted mean HRQoL values (EQ-5D and VAS) for all values of the SEP score.

As an alternative analysis of variation in HRQoL, we applied the concentration index (CI). The CI measures the degree of socioeconomic inequality in HRQoL [47]. The CI's range is [-1,1], with the value 0 indicating perfect equality. A positive (negative) value indicates that the distribution of HRQoL is ‘pro-high SEP’ (‘pro-low SEP’) [48]. We compared CIs using the SEP score as the variable from which to rank individuals, with education and income.

### Sensitivity analyses

We performed age-stratified analyses to assess whether the education and income weights derived for the composite SEP score differed across age groups. These analyses were conducted by running separate adjacent-category logistic regression analyses stratified by age groups (40–49; 50–65; and 66–79). Sex-specific analyses were also conducted, as well as analyses including only respondents who were currently in the labour force (full or part time). Lastly, we tested equivalising the household income variable with marital status.

We randomly split the sample in equal halves (referred to as Subsamples 1 and 2), before rerunning the adjacent-category logistic regression as in Eq. 1 on both samples. Next, we conducted the same procedure as in Eq. 2, using regression coefficients from Subsample 1, generating an alternative composite SEP score. With OLS regression, we tested how well the composite SEP score with weights from Subsample 1 performed in predicting HRQoL (EQ-5D and VAS) in Subsample 2. We assessed how these estimates (composite SEP score coefficients and the R^2^) differed from the analyses on HRQoL run on the full sample.

All statistical analyses were performed with Stata© version 15.1 (Stata Corporation, College Station, Texas).

## Results

### Descriptive statistics

Table 1 reports respondent characteristics. Given that this is a community sample, respondents were healthy in general, with a mean EQ-5D value of 0.89 and a mean VAS score of 0.76. Among them, 28.6% can be classified as in ‘full health’, i.e., they reported ‘no problems’ in all five EQ-5D-5L dimensions. The proportion of the sample with tertiary level education was larger than in the corresponding age group from the Norwegian population (50.0% compared to 33.1%, respectively) [49].

**Table 1:** Sample characteristics

**Variables**	**Female**		**Male**		**Total**	
	**Mean/%**	**N**	**Mean/%**	**N**	**Mean/%**	**N**
Age, mean (SD)	56.2 (10.36)	10,661	56.5 (10.44)	9,661	56.3 (10.40)	20,322
Education level						
Primary education < 10 yrs	22.6	2,375	21.4	2,033	22.0	4,408
Upper secondary/vocational	25.5	2,681	30.6	2,915	27.9	5,596
Undergraduate degree	18.0	1,890	21.4	2,040	19.6	3,930
Postgraduate degree	33.9	3,561	26.6	2,532	30.4	6,093
Income						
Low	25.2	2,546	16.3	1,542	20.9	4,088
Lower middle	30.0	3,033	28.6	2,700	29.3	5,733
Upper middle	22.4	2,265	26.0	2,462	24.2	4,727
High	22.3	2,257	29.1	2,753	25.6	5,010
Subjective SEP						
Very low/low	7.6	784	6.2	587	6.9	1,371
Middle	54.5	5,638	47.1	4,449	51.0	10,087
Fairly high	31.9	3,295	38.7	3,657	35.1	6,952
Very high	6.1	627	8.1	761	7.0	1,388
HRQoL: EQ-5D-5L						
Full health (11111), %	24.9	2,560	32.6	3,034	28.6	5,594
Mean	0.88	10,275	0.90	9,322	0.89	19,597
(SD)	(0.11)		(0.10)		(0.11)	
HRQoL: VAS score	0.76	10,472	0.77	9,500	0.76	19,972
(SD)	(0.17)		(0.15)		(0.16)	

The undergraduate and postgraduate education levels correspond to university education up to four years and university education of four years or more, respectively; mean value for EQ-5D-5L measured by WePP: Western Preference Pattern. SD: standard deviation; HRQoL: health-related quality of life; VAS: visual analogue scale, converted into a [0–1] interval

### Weights for the composite SEP score

Table 2 displays the adjacent-category logistic regression output. Education was the main driver for subjective SEP, as demonstrated by the clear increase in the size of the education coefficient for each level change, particularly so for a postgraduate degree. For income, there was a non-linear increase in the size of the coefficients, with the highest income coefficient being thrice as large as the upper-middle income coefficient.

**Table 2:** Adjacent-category logistic regression on subjective SEP: weights for the composite SEP score based on education and income

	**Coefficient** **(SE)**
**Education level**	
Primary education < 10 yrs	Ref
Upper secondary/ vocational	0.141*** (0.034)
Undergraduate degree	0.697*** (0.038)
Postgraduate degree	1.293*** (0.037)
**Income**	
Low income	Ref
Lower-middle income	0.193*** (0.034)
Upper-middle income	0.261*** (0.037)
High income	0.822*** (0.039)
**Demographic characteristics**	
Age (years)	0.020*** (0.001)
Male	0.270*** (0.023)
Constant 1	0.180** (0.083)
Constant 2	-2.543*** (0.142)
Constant 3	-4.233*** (0.194)
*Observations*	*18,988*
*AIC*	*37,550*
*Pseudo R*^*2*^	*0.0886*

^***^ p < 0.01, ** p < 0.05, * p < 0.1. The undergraduate and postgraduate education levels correspond to university education up to four years, and university education of four years or more, respectively; SE: Standard errors in parentheses; Male: binary variable: 0 = female; 1 = male; AIC: Akaike’s Information Criterion

The weights derived were used to generate the composite SEP score (Eq. 2). The rescaled and rounded SEP score is reported in a ‘4X4 SEP table’ (Table 3). This table indicates that the observed non-linearities reported in Table 2 are reinforced when combining education and income levels.

**Table 3:** ‘4X4 SEP’ table, combining education and income levels

	**Income**			
	Low	Lower-middle	Upper-middle	High
**Education**				
Primary education < 10 yrs	1	2	2	4
Upper secondary/vocational	2	2	3	5
Undergraduate degree	4	5	5	7
Postgraduate degree	7	7	8	10

Predicted socioeconomic position (SEP) score based on all combinations of education and income levels

### Predicting variation in HRQoL with the composite SEP score

Table 4 provides the results from the OLS regression of the composite SEP score on both HRQoL measures (EQ-5D and VAS). A one-unit increase in SEP is associated with an average increase of 0.006 in the case of EQ-5D and 0.010 for VAS. Comparing the results with OLS regression of education and income separately led to a similar model fit based on the R^2^ (output not shown).

**Table 4:** Ordinary least squares regression on HRQoL (EQ-5D-5L values and VAS scores) with the composite SEP score as the independent variable

	**EQ-5D**	**VAS**
	**Coefficient (Robust SE)**	**Coefficient (Robust SE)**
Composite SEP score	0.006*** (0.000)	0.010*** (0.000)
Age (yrs)	0.001*** (0.000)	< 0.001*** (0.000)
Male	0.022*** (0.002)	0.002 (0.002)
Constant	0.820*** (0.005)	0.689*** (0.008)
*Observations*	*18,761*	*19,119*
*R*^*2*^	*0.0369*	*0.0338*

^***^ p < 0.01, ** p < 0.05, * p < 0.1; HRQoL was measured by the WePP: Western Preference Pattern for EQ-5D-5L; and the VAS: visual analogue scale; Male: binary variable: 0 = female; 1 = male; robust standard errors (SE) in parentheses

**Fig. 2:**
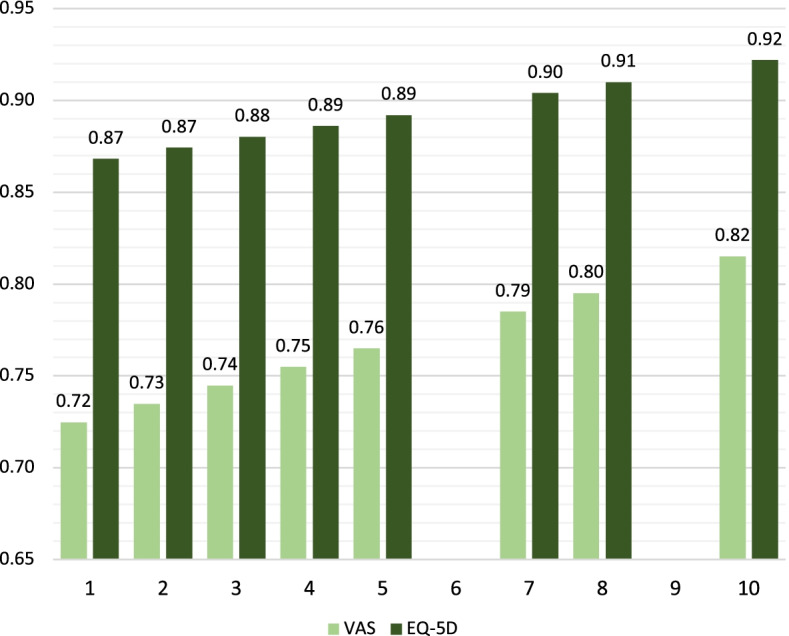
Age-adjusted mean EQ-5D values and VAS scores by composite SEP score. Mean VAS scores (left bars) and EQ-5D values (right bars) for each SEP score. SEP scores 6 and 9 are empty due to no data for these SEP score values. SEP: socioeconomic position

Figure 2 presents age-adjusted mean EQ-5D values and VAS scores by SEP score levels. There was a clear linear increase in the reported HRQoL scores as the SEP values increased from 1 to 10, with EQ-5D values consistently higher than the VAS scores. The gradient was steeper for VAS (range: 0.72–0.82) than for EQ-5D (range: 0.87–0.92).

The concentration indices of HRQoL are reported in Additional file 1. The CIs using the SEP score were 0.020 and 0.040 for EQ-5D and VAS, respectively. The CIs using education and income were slightly larger. The positive values of the CI indicate that better HRQoL were concentrated among respondents with a higher SEP.

### Sensitivity analyses

Age-group stratified analyses of the determinants of subjective SEP indicated that the importance of education increased with age, whereas income became less important with age (Additional file 2). In terms of sex differences, the second-lowest education level was not statistically different from the reference among women. The patterns are the same as in the main model, with increasing coefficient sizes for each level increase in education and income (Additional file 3). Restricting the sample to respondents who stated being actively employed led to similar results as the main model, except for a non-significant upper secondary education coefficient (Additional file 4). Analysing household income equivalised for marital status did not lead to substantially different estimates. This output was therefore not included.

Using the composite SEP score with weights generated from Subsample 1 (Additional file 5) to predict both EQ-5D and VAS on Subsample 2, the coefficients remained similar and the change in R^2^ was marginal (Additional file 6).

## Discussion

This paper has proposed a composite SEP score by modelling individuals’ subjective SEP based on four education and four income levels. The derived weights demonstrated how education and income influenced subjective SEP. There were non-linearities in determining subjective SEP, with greater importance placed on the higher education and income levels. These non-linearities became more evident when combining the different education and income levels, indicating that higher levels of education and income reinforced each other. The score was used to estimate inequalities in HRQoL based on combinations of education and income, and for each level of the composite SEP score. We found a clear gradient in HRQoL, with a linear increase from the bottom to the top of the score.

The proposed composite SEP score was derived from a measure of *subjective* SEP. This is in line with research recognising the added value of supplementing objective measures with subjective measures [12, 13, 50]. We contribute to the literature with a composite SEP score that captured both subjective and objective aspects of SEP, in which the objective indicators (education and income) estimated the subjective component (subjective SEP). The subjective SEP measure applied here differs from the more commonly used MacArthur scale of subjective social status [11]. Whereas the MacArthur scale is framed in terms of education, occupation and income, the subjective SEP measure is closely tied to occupation. Moreover, it should not be confused with occupational *prestige*, since the measure applied in this paper captures individuals’ perception of their own occupation’s social status, not the society’s judgement of the status of specific occupations [35].

The composite SEP score was estimated by education and income. Occupational category was not included in the estimation because we assumed that its influence on SEP was captured in its intermediate role between education (the determinant of occupation) and income (the reward of occupation). Moreover, in contrast to education (years) and income (money), the occupational categories are not as easily hierarchically ordered, in line with the arguments presented by Braveman et al. [8]. Education and income are more often consistently measured and available across different surveys and registers [27]. Besides, social standing derived from occupation is arguably more context dependent: a fisherman’s standing is likely judged higher in his local community than in the big city. We therefore followed Freeman et al. in omitting occupation [27].

Furthermore, the role of parental and early-life SEP when determining adult SEP must be acknowledged, the importance of which is consistently found to be substantial in the literature: children born to parents with higher SEP are more likely to prosper both in terms of socioeconomic achievements and in terms of health (e.g., [34, 51]). These factors are essential in the understanding of SEP.

The observed non-linear relationship of education and income in determining subjective SEP was evident from Table 3, with large marginal increases in subjective SEP from the highest education level, regardless of income. These non-linearities are likely to have different explanations. For example, Norway has a relatively egalitarian income distribution and a generous welfare state, which is likely to contribute to income being of less importance for most people. For the richest, however, income could matter more for SEP, potentially because social success can be signalled through various types of conspicuous consumption [52], such as living in a posh neighbourhood.

Age-stratified analyses added additional insights on cohort effects. Education appeared to matter more for the older age groups, whereas the size of the income coefficients decreased with age (Additional file 1). This could imply that education was a relatively stronger determinant of subjective SEP for those who did have higher education in the oldest age group (66–79). Indeed, the share of people opting for higher education has dramatically increased over the past generations, suggesting that higher education was more important for SEP when it was more of a privilege for the few. Cohort effects are also relevant in the case of sex differences (Additional file 3), in that women constitute a larger share of those taking higher education. The non-significant upper secondary/vocational coefficient in women could reflect that taking higher education is more important for women’s SEP than for men’s.

The relative importance of education and income in predicting SEP will likely vary between countries [53, 54]. If a similar analysis had been performed in a country with larger income inequalities than Norway, there would likely be starker differences between all the income categories, not only the top one as in this sample. Therefore, it is important to consider international differences in the relative importance of socioeconomic factors as determinants of SEP.

Our results indicated that the composite SEP score predicted considerable variation in HRQoL. Although there was no difference in the predictive power of the composite SEP score model compared to analysing education and income separately, it is arguably a more convenient way to calculate the combined impact of education and income on health inequalities, rather than conducting separate analyses [17, 55]. Moreover, the composite SEP score allowed us to demonstrate a linear increase in the age-adjusted HRQoL value by SEP score level for both the EQ-5D and the VAS (Fig. 2). This indicates a clear social gradient in both HRQoL measures, a message that would be hard to communicate with separate indicators.

The use of an alternative measure of inequality, the concentration index, suggested that inequalities in HRQoL are concentrated among higher-SEP groups, although the degree of inequality is relatively low (Additional file 1). The CIs of education and income were slightly larger than those of the composite SEP score, which could suggest that the combination of education and income somewhat compensates for differential variation in these two SEP variables. The order of magnitude of these results is comparable to other studies investigating inequalities in HRQoL [48, 56].

For the split-sample analyses, the estimates from the OLS analysis with the alternative composite SEP score (Additional file 6) did not differ greatly from the results in Table 4. This suggests that our estimates were internally valid.

### Strengths and limitations

The key contribution of the current paper is that we provide a new application of a regression-based method for developing a composite SEP score with empirically derived education and income weights along a [1-10] SEP scale. Since education is grouped into the standard four levels, and income is approximately grouped into quartiles, our proposed approach can be replicated in any cohort study that collects these data, on any health outcome. Second, we provide new insights into the relative importance of different education and income levels as sources of SEP. Third, we have shown how SEP in the form of a composite score can be applied in analyses of health inequalities.

Some limitations should be acknowledged. First, the sample consists of respondents aged 40–79, leaving out the younger segment of the adult population. Second, since the subjective SEP measure targets people in the labour force, there is a risk that respondents who did not work at the time of the survey did not answer the question ‘correctly’, although the question specified that those who were not currently working should think about their latest occupation, assuming that an individual’s *previous* occupation is important for their current SEP. Sensitivity analyses indicated that including only currently employed respondents did not dramatically differ from the main results. Third, although a missing rate of observations of 4.5% is relatively small, there could still be systematic differences between the included and excluded shares of the sample. Missing value analysis indicated that those not reporting education or income were older and had a larger proportion of women compared to the full sample. We therefore cannot rule out that our results could underestimate inequalities, since older respondents would be more likely to report a lower HRQoL.

## Conclusions

Our results suggest that a composite SEP score should be considered when studying social inequalities in health. We have proposed a model for a composite SEP score that predicts individuals’ SEP based on empirically weighted combinations of education and income levels, which identified a clear social gradient in HRQoL. This approach could be used when data on education and income are collected, either in cohort studies or through registers, potentially predicting the SEP of the entire population. The weights derived in this paper are relevant in a Norwegian context. Research from other countries is needed to compare the relative importance of education and income as determinants of SEP across countries, and to investigate how a composite SEP score would predict health inequalities in other institutional contexts.

## Supplementary Information

**Additional file 1.** Concentration index of EQ-5D and VAS

**Additional file 2.** Adjacent-category logisticregression on subjective social status: weights for composite SEP score,stratified by age groups.

**Additional file 3.** Adjacent-category logistic regression onsubjective SEP: stratified by sex.

**Additional file 4.** Adjacent-category logistic regression onsubjective SEP, including only currently employed respondents (full or parttime).

**Additional file 5.** Adjacent-category logistic regression onsubjective social status: weights for composite SEP score with sample randomlysplit in two.

**Additional file 6.** Ordinary least squares regression analysis to test internalvalidity.

## Data Availability

The data that support the findings of this study are available from The Tromsø Study but restrictions apply to the availability of these data, which were used under license for the current study, and so are not publicly available. In order to get access to the data on which the present study is based, permission from the Tromsø Study is required. The Data and Publication Committee of the Tromsø Study evaluates all applications for access to data, and upon approval of application, an agreement is made between The Tromsø Study and the project manager of the project in question. Questions regarding access to data may be directed towards tromsous@ism.uit.no.

## Abbreviations

CI: Concentration Index
EQ-5D: EuroQol Five-dimensional Measure of Health-related Quality of Life
HRQoL: Health-Related Quality of Life
OLS: Ordinary Least Squares
SD: Standard Deviation
SE: Standard Error
SEP: Socioeconomic Position
VAS: Visual Analogue Scale
WePP: Western Preference Pattern
