# Supplementary material for: Combining education and income into a socioeconomic position score for use in studies of health inequalities
Source: BMC Public Health. 2022 May 13;22:969. doi: 10.1186/s12889-022-13366-8 (PMC9107133; doi:10.1186/s12889-022-13366-8)
Supplement: Supplementary file 1 — Additional file 1. Concentration index of EQ-5D and VAS [file 12889_2022_13366_MOESM1_ESM.docx]

Additional file 1: Concentration index of EQ-5D and VAS

|  | **SEP score CI**  (SE) | **Education CI**  (SE) | **Income CI**  (SE) |
| --- | --- | --- | --- |
| **EQ-5D** | 0.020  (0.001) | 0.022  (0.000) | 0.023  (0.000) |
| **VAS** | 0.040  (0.001) | 0.047  (0.001) | 0.043  (0.001) |

*Note:* *SEP*: socioeconomic position; *CI*: concentration index; HRQoL was measured by the *WePP*: Western Preference Pattern for EQ-5D-5L; and *VAS*: visual analogue scale; standard errors (SE) in parentheses.
