# Supplementary material for: Combining education and income into a socioeconomic position score for use in studies of health inequalities
Source: BMC Public Health. 2022 May 13;22:969. doi: 10.1186/s12889-022-13366-8 (PMC9107133; doi:10.1186/s12889-022-13366-8)
Supplement: Supplementary file 2 — Additional file 2. Adjacent-category logisticregression on subjective social status: weights for composite SEP score,stratified by age groups. [file 12889_2022_13366_MOESM2_ESM.docx]

Additional file 2: Adjacent-category logistic regression on subjective social status: weights for composite SEP score, stratified by age groups.

|  | **Age groups** | | |
| --- | --- | --- | --- |
|  | **40-49** | **50-65** | **66+** |
|  | **Coefficients (SE)** | **Coefficients (SE)** | **Coefficients (SE)** |
| **Education level** | | | |
| Primary/lower secondary school | Ref. | Ref. | Ref. |
| Upper secondary/ vocational school | 0.051  (0.075) | 0.085*  (0.050) | 0.319***  (0.068) |
| Undergraduate degree | 0.606***  (0.079) | 0.630***  (0.054) | 0.860***  (0.079) |
| Post-graduate degree | 1.128***  (0.077) | 1.271***  (0.054) | 1.428***  (0.081) |
| **Income level** | | | |
| Low income | Ref. | Ref. | Ref. |
| Lower-middle income | 0.421***  (0.072) | 0.140***  (0.053) | 0.087  (0.056) |
| Upper-middle income | 0.388***  (0.071) | 0.231***  (0.054) | 0.306***  (0.083) |
| High income | 1.058***  (0.073) | 0.718***  (0.056) | 0.733***  (0.106) |
| **Demographic characteristic** | | | |
| Male | 0.232***  (0.039) | 0.247***  (0.034) | 0.404***  (0.052) |
|  | | | |
| Constant 1 | 0.841***  (0.087) | 1.441***  (0.062) | 1.820***  (0.080) |
| Constant 2 | -1.621***  (0.139) | -1.348***  (0.085) | -1.247***  (0.077) |
| Constant 3 | -3.328***  (0.198) | -3.009***  (0.128) | -2.980***  (0.142) |
| *Observations* | *6,228* | *8,800* | *3,960* |
| *AIC* | *12695* | *17300* | *7516* |
| *Pseudo R^2^* | *0.0905* | *0.0884* | *0.0901* |

*Note:* *** p<0.01, ** p<0.05, * p<0.1; the undergraduate and post-graduate education levels correspond to university education up to four years, and university education of four years or more, respectively; *Male*, binary variable: 0=female; 1=male; *SEP*: socioeconomic position; *AIC,* Akaike’s Information Criterion; *SE,* standard errors in parentheses.
