# Supplementary material for: Combining education and income into a socioeconomic position score for use in studies of health inequalities
Source: BMC Public Health. 2022 May 13;22:969. doi: 10.1186/s12889-022-13366-8 (PMC9107133; doi:10.1186/s12889-022-13366-8)
Supplement: Supplementary file 3 — Additional file 3. Adjacent-category logistic regression onsubjective SEP: stratified by sex. [file 12889_2022_13366_MOESM3_ESM.docx]

Additional file 3: Adjacent-category logistic regression on subjective SEP: stratified by sex

|  | **Women** | **Men** |
| --- | --- | --- |
|  | **Coefficient**  **(SE)** | **Coefficient**  **(SE)** |
| **Education level** | | |
| Primary education <10 yrs | Ref. | Ref. |
| Upper secondary/ vocational school | 0.037  (0.050) | 0.220***  (0.048) |
| Undergraduate degree | 0.657***  (0.055) | 0.722***  (0.052) |
| Post-graduate degree | 1.255***  (0.053) | 1.326***  (0.054) |
| **Household income level** | | |
| Low income | Ref. | Ref. |
| Lower-middle income | 0.164***  (0.045) | 0.243***  (0.052) |
| Upper-middle income | 0.197***  (0.050) | 0.351***  (0.055) |
| High income | 0.669***  (0.052) | 0.989***  (0.058) |
| **Demographic characteristics** | | |
| Age (yrs) | 0.017***  (0.002) | 0.023***  (0.002) |
|  | | |
| Constant 1 | 0.546***  (0.120) | 0.056  (0.118) |
| Constant 2 | -2.300***  (0.204) | -2.526***  (0.200) |
| Constant 3 | -3.824***  (0.277) | -4.380***  (0.276) |
| *Observations* | *9,792* | *9,196* |
| *AIC* | *19182* | *18332* |
| *Pseudo R^2^* | *0.0802* | *0.0936* |

*Note:* *** p<0.01, ** p<0.05, * p<0.1; the undergraduate and post-graduate education levels correspond to university education up to four years, and university education of four years or more, respectively; *SEP*: socioeconomic position; *AIC,* Akaike’s Information Criterion; *SE,* standard errors in parentheses.
