# Supplementary material for: Combining education and income into a socioeconomic position score for use in studies of health inequalities
Source: BMC Public Health. 2022 May 13;22:969. doi: 10.1186/s12889-022-13366-8 (PMC9107133; doi:10.1186/s12889-022-13366-8)
Supplement: Supplementary file 4 — Additional file 4. Adjacent-category logistic regression onsubjective SEP, including only currently employed respondents (full or parttime). [file 12889_2022_13366_MOESM4_ESM.docx]

Additional file 4: Adjacent-category logistic regression on subjective SEP, including only currently employed respondents (full or part time)

|  | **Coefficient**  **(SE)** |
| --- | --- |
| **Educational level** | |
| Primary education <10 yrs | Ref. |
| Upper secondary/ vocational school | 0.017  (0.046) |
| Undergraduate degree | 0.604***  (0.049) |
| Post-graduate degree | 1.220***  (0.048) |
| **Income** | |
| Low income | Ref. |
| Lower-middle income | 0.261***  (0.052) |
| Upper-middle income | 0.283***  (0.052) |
| High income | 0.892***  (0.053) |
| **Demographic characteristics** | |
| Age (yrs) | 0.017***  (0.002) |
| Male | 0.252***  (0.027) |
|  | |
| Constant 1 | 0.427***  (0.113) |
| Constant 2 | -2.317***  (0.191) |
| Constant 3 | -4.063***  (0.257) |
| *Observations* | *13,371* |
| *AIC* | *26425* |
| *Pseudo R^2^* | *0.0931* |

*Note:* *** p<0.01, ** p<0.05, * p<0.1; the undergraduate and post-graduate education levels correspond to university education up to four years, and university education of four years or more, respectively; *Male*, binary variable: 0=female; 1=male; *SEP*: socioeconomic position; *AIC,* Akaike’s Information Criterion; *SE,* standard errors in parentheses
