# Supplementary material for: Combining education and income into a socioeconomic position score for use in studies of health inequalities
Source: BMC Public Health. 2022 May 13;22:969. doi: 10.1186/s12889-022-13366-8 (PMC9107133; doi:10.1186/s12889-022-13366-8)
Supplement: Supplementary file 5 — Additional file 5. Adjacent-category logistic regression onsubjective social status: weights for composite SEP score with sample randomlysplit in two. [file 12889_2022_13366_MOESM5_ESM.docx]

Additional file 5: Adjacent-category logistic regression on subjective social status: weights for composite SEP score with sample randomly split in two

|  | **Subsample 1** | **Subsample 2** | ***Original sample***  ***(from Table 2)*** |
| --- | --- | --- | --- |
|  | **Coefficient**  **(SE)** | **Coefficient**  **(SE)** | ***Coefficient***  ***(SE)*** |
| **Educational level** | | | |
| Primary/lower secondary school | Ref. | Ref. | *Ref.* |
| Upper secondary/ vocational school | 0.110**  (0.048) | 0.173***  (0.049) | *0.141****  *(0.034)* |
| Undergraduate degree | 0.647***  (0.053) | 0.748***  (0.054) | *0.697****  *(0.038)* |
| Post-graduate degree | 1.252***  (0.052) | 1.336***  (0.053) | *1.293****  *(0.037)* |
| **Income** | | | |
| Low income | Ref. | Ref. | *Ref.* |
| Lower-middle income | 0.218***  (0.048) | 0.166***  (0.049) | *0.193****  *(0.034)* |
| Upper-middle income | 0.307***  (0.051) | 0.213***  (0.052) | *0.261****  *(0.037)* |
| High income | 0.862***  (0.054) | 0.779***  (0.055) | *0.822****  *(0.039)* |
| **Demographic characteristics** | | | |
| Age (yrs) | 0.020***  (0.002) | 0.021***  (0.002) | *0.020****  *(0.001)* |
| Male | 0.270***  (0.032) | 0.272***  (0.032) | *0.270****  *(0.023)* |
|  | | | |
| Constant 1 | 0.177  (0.117) | 0.183  (0.119) | *0.180***  *(0.083)* |
| Constant 2 | -2.498***  (0.198) | -2.588***  (0.203) | *-2.543****  *(0.142)* |
| Constant 3 | -4.202***  (0.272) | -4.266***  (0.278) | *-4.233****  *(0.194)* |
| *Observations* | *9,504* | *9,843* | *18,988* |
| *AIC* | *18,892* | *18,676* | *37,550* |
| *Pseudo R^2^* | *0.0882* | *0.0892* | *0.0886* |

*Note:* *** p<0.01, ** p<0.05, * p<0.1; the undergraduate and post-graduate education levels correspond to university education up to four years, and university education of four years or more, respectively; *Male*: binary variable: 0=female; 1=male; *SEP*: socioeconomic position; *AIC*: Akaike’s Information Criterion; *SE*: standard errors in parentheses. Estimates from Table 2 in the right column.
