# Supplementary material for: Combining education and income into a socioeconomic position score for use in studies of health inequalities
Source: BMC Public Health. 2022 May 13;22:969. doi: 10.1186/s12889-022-13366-8 (PMC9107133; doi:10.1186/s12889-022-13366-8)
Supplement: Supplementary file 6 — Additional file 6. Ordinary least squares regression analysis to test internalvalidity. [file 12889_2022_13366_MOESM6_ESM.docx]

Additional file 6: Ordinary least squares regression analysis to test internal validity

|  | **EQ-5D-5L** | | **VAS** | |
| --- | --- | --- | --- | --- |
|  | **Subsample 2** | ***Full sample (from Table 4)*** | **Subsample 2** | ***Full sample (from Table 4)*** |
|  | **Coefficients (Robust SE)** | ***Coefficients (Robust SE)*** | **Coefficients (Robust SE)** | ***Coefficients (Robust SE)*** |
| Composite SEP score | 0.006***  (0.000) | *0.006****  *(0.000)* | 0.010***  (0.001) | *0.010****  *(0.000)* |
| Age (yrs) | 0.001***  (0.000) | *0.001****  *(0.000)* | 0.001  (0.000) | *<0.001****  *(0.000)* |
| Male | 0.023***  (0.002) | *0.022****  *(0.002)* | 0.003  (0.003) | *0.002*  *(0.002)* |
| Constant | 0.819***  (0.008) | *0.820****  *(0.005)* | 0.684***  (0.011) | *0.689****  *(0.008)* |
| *Observations* | *9,392* | *18,761* | *9,821* | *19,119* |
| *R^2^* | *0.0385* | *0.0369* | *0.0328* | *0.0338* |

*Note:* *** p<0.01, ** p<0.05, * p<0.1; *WePP*, Western Preference Pattern for EQ-5D-5L; *VAS*, visual analogue scale; *Male*, binary variable: 0=female; 1=male; *SEP*: socioeconomic position; robust standard errors (*SE*) in parentheses. Split-sample test using weights generated from Subsample 1, on Subsample 2, with EQ-5D and VAS as dependent variables, in the left and right panels, respectively.
